# Defining the Balance between Regeneration and Pathological Ossification in Skeletal Muscle Following Traumatic Injury

**DOI:** 10.3389/fphys.2017.00194

**Published:** 2017-04-03

**Authors:** Owen G. Davies, Yang Liu, Darren J. Player, Neil R. W. Martin, Liam M. Grover, Mark P. Lewis

**Affiliations:** ^1^School of Sport, Exercise and Health Sciences, Loughborough UniversityLoughborough, UK; ^2^School of Chemical Engineering, University of BirminghamBirmingham, UK; ^3^Wolfson School of Mechanical and Manufacturing Engineering, Loughborough UniversityLoughborough, UK; ^4^National Centre for Sport and Exercise Medicine, Arthritis Research UK Centre for Sport, Exercise and Osteoarthritis, School of Sport, Exercise and Health Sciences, Loughborough UniversityLoughborough, UK

**Keywords:** heterotopic ossification, hypoxia-inducible factor 1, endochondral ossification, macrophage polarization, bone morphogenetic proteins, vascular endothelial growth factors, satellite cells, skeletal muscle

## Abstract

Heterotopic ossification (HO) is characterized by the formation of bone at atypical sites. This type of ectopic bone formation is most prominent in skeletal muscle, most frequently resulting as a consequence of physical trauma and associated with aberrant tissue regeneration. The condition is debilitating, reducing a patient's range of motion and potentially causing severe pathologies resulting from nerve and vascular compression. Despite efforts to understand the pathological processes governing HO, there remains a lack of consensus regarding the micro-environmental conditions conducive to its formation, and attempting to define the balance between muscle regeneration and pathological ossification remains complex. The development of HO is thought to be related to a complex interplay between factors released both locally and systemically in response to trauma. It develops as skeletal muscle undergoes significant repair and regeneration, and is likely to result from the misdirected differentiation of endogenous or systemically derived progenitors in response to biochemical and/or environmental cues. The process can be sequentially delineated by the presence of inflammation, tissue breakdown, adipogenesis, hypoxia, neo-vasculogenesis, chondrogenesis and ossification. However, exactly how each of these stages contributes to the formation of HO is at present not well understood. Our previous review examined the cellular contribution to HO. Therefore, the principal aim of this review will be to comprehensively outline changes in the local tissue micro-environment following trauma, and identify how these changes can alter the balance between skeletal muscle regeneration and ectopic ossification. An understanding of the mechanisms governing this condition is required for the development and advancement of HO prophylaxis and treatment, and may even hold the key to unlocking novel methods for engineering hard tissues.

## Background

Heterotopic ossification (HO) is defined as the formation of mature bone at atypical sites, such as skeletal muscle or tendon. Patients with HO will experience a range of clinical problems resulting from the formation of extra-skeletal hard tissue, including pain, joint ankylosis, and neurovascular entrapment, as well as problems with prosthetic limb fitting. Current prophylaxis and treatments have varying levels of success and include the use of non-steroidal anti-inflammatories (NSAIDs), bisphosphonates, and single dose radiotherapy. A thorough discussion of the benefits and drawbacks of these clinical methods can be found in a previous review (Davies et al., 2015). Currently, the underlying mechanisms governing acquired HO are not well understood, with the osteoinductive action of certain BMPs, implicated in genetic forms of the condition, such as fibrodysplasia ossificans progressiva (FOP), likely to be only partly responsible. A prevailing view is that acquired HO occurs as a consequence of aberrant muscle repair resulting from a chronic inflammatory environment. Although, the exact switch defining the balance between muscle repair and HO is likely to be complex and multifaceted. This complexity arises from the overall scale of the local and systemic response to trauma, as well as the differential action of pro- and anti-inflammatory cytokines on the heterogeneous population of cells present at the trauma site. This includes cells of different origins and states of commitment, such as endogenous skeletal muscle cells and migratory multipotent progenitors, as well as pericytes and endothelial cells derived from the local vasculature. With this in mind, the aim of the review is to identify how chronic inflammation can alter the balance between skeletal muscle regeneration and pathological outcomes such as HO, in particular detailing how inflammatory dysregulation may contribute to the misdirected differentiation of cells localized at the wound bed. We will also place this in context with the current understanding of the sequential series of events leading to the formation of HO to better understand this debilitating phenomenon.

## Inflammation

**Figure 1:**
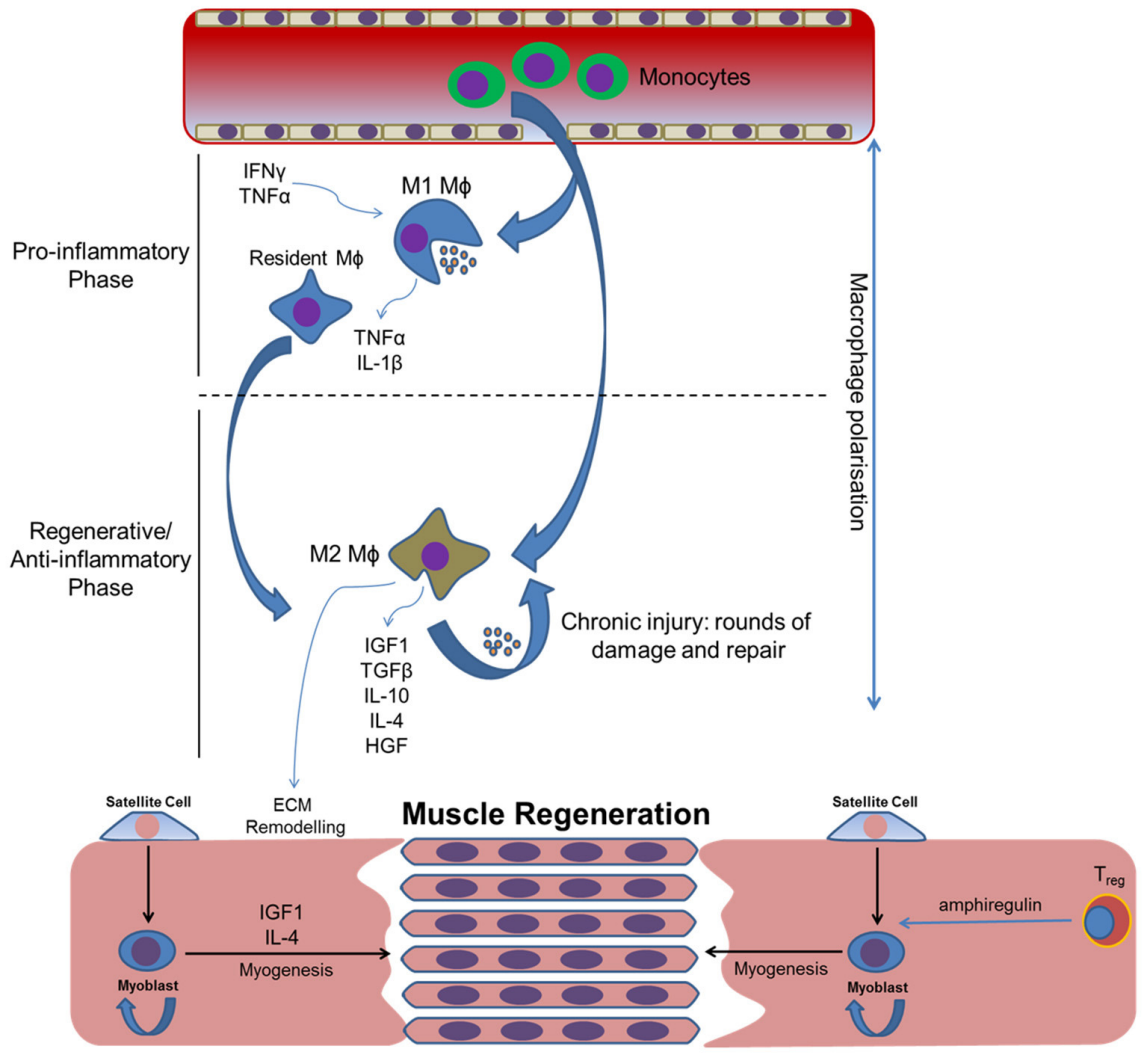
**How inflammatory cytokines and subsequent macrophage polarization may influence skeletal muscle regeneration following traumatic injury**. Macrophage polarization coordinates the initial pro-inflammatory and subsequent regenerative/anti-inflammatory phases required for the removal of local debris and the regeneration of tissue, respectively. Inflammatory dysregulation will alter the balance of these phases, compromising effective tissue repair and potentially leading to tissue fibrosis and subsequent HO. Circular arrows indicate self-renewal.

Inflammation is known to play an important and multifaceted role in directing cell recruitment, vascularization, and cartilage remodeling during bone repair. Therefore, it has been hypothesized that persistent inflammatory dysregulation resulting from severe traumas such as military blast injuries may negatively affect wound healing and be conducive to extra-skeletal ossification in soft tissues (Hahm et al., 2011). Physical injury triggers an inflammatory cascade, which is characterized by the rapid and sequential invasion of leukocytes that persists throughout muscle repair, regeneration and growth. Studies detailing the inflammatory response of this tissue to trauma have shown that the first inflammatory cells recruited to the site of muscle injury are the neutrophils and T helper cells (Th) (Loell and Lundberg, 2010). The primary role of these cells is to remove tissue debris from the damaged area and contribute to the activation of resident satellite cells to promote muscle regeneration. The migration of neutrophils and Th is followed by the sequential infiltration of other inflammatory cell types, with macrophages dominating after approximately 24 h (McClung et al., 2007). The invasion of macrophages functions to remove necrotic tissue, as well as facilitating myoblast proliferation and differentiation to promote muscle repair. However, macrophages are extremely plastic cells, with previous studies showing that the exact function of these cells is dependent on the local tissue environment (Novak et al., 2015). Since severe traumas linked with HO (e.g., blast trauma or traumatic brain injury) can cause unrecognizable damage to local and surrounding tissues, this raises the question of how macrophage plasticity influences the balance between skeletal muscle repair and HO. At the most basic level macrophages can be split into two subsets: M1 “pro-inflammatory” and M2 “anti-inflammatory” cells, with the two subtypes having distinct roles during tissue regeneration. In human muscle, M1 macrophages are predominantly associated with satellite cell activation and proliferation, whereas M2 macrophages have an increased presence during terminal myogenesis (Saclier et al., 2013). Both subtypes are known to coexist in chronically injured muscle, yet their effects on tissue repair and homeostasis are negligible (Rigamonti et al., 2014). At present no studies have comprehensively detailed the inflammatory events that occur within chronic muscle wounds, with the majority of research confined to models of mild/moderate trauma. As a consequence, the temporal roles of M1 and M2 macrophages in this process remain unclear, and the effects of inflammatory events such as macrophage polarization on HO are not fully understood (Figure 1). Advancing our current understanding of the association between inflammatory dysregulation and ectopic ossification will be of considerable benefit for identifying novel prophylactics and treatments for both genetic and acquired forms of HO, as well as for the formulation of new regenerative therapies for bone tissue engineering. We shall now direct our attention to the expanding number of proteomic studies that have sought to profile cytokines within the post-trauma environment, and the effects these cytokines may have on HO.

It has long been established that severe injuries such as blast trauma, traumatic brain injury (TBI) and spinal cord injury (SCI) are linked with HO, and that factors present in the local wound bed, effluent, or released into the circulation following these injuries may have positive relationship with extra-skeletal ossification (Hawksworth et al., 2009). A popular approach has been to proteomically analyse the wound site to identify the roles that humoral factors have in the induction or enrichment of the HO environment. Current proteomic studies face several technical limitations principally concerning the maximum resolution of a given technique or unstandardized methods of sample preparation, as well as uncontrollable variables such as the timing of sample acquisition, scale of trauma, and the type of trauma (blast, TBI, SCI, burn etc.). Nonetheless, despite these limitations such studies may prove highly valuable and may be applied for the development of much needed diagnostic tools. The development of such tools may someday enable clinicians to provide a quantifiable prediction of a patient's risk of developing HO, as well as other negative outcomes associated with blast trauma such as wound dehiscence (Forsberg et al., 2008). Current research has identified that there are at least 14 proteins exclusive to the serum of TBI patients that have a direct binding affinity with human osteoprogenitors *in vitro* (Cadosch et al., 2009b). However, full bioinformatic characterization of these proteins is required before any conclusions can be made regarding their effects on muscle regeneration and HO. Significantly, in several rat models of induced TBI, proteomic analysis of the local serum has identified many of the same cytokines and growth factors present during fracture repair, such as TGF-β, insulin-like growth factor (IGF), IL-1, IL-6 and BMPs (Pasinetti et al., 1993; Kim et al., 1996; Jackson et al., 2012). A subsequent human study that collected and analyzed the serum and wound exudate of victims of penetrating combat-related traumas found increased levels of a number of interleukins, as well as the presence of monocyte chemoattractant protein-1 (MCP-1), interferon gamma-induced protein 10 (IP-10) and macrophage inflammatory protein alpha (MIP-α) (Evans et al., 2012). Many of the cytokines identified in the local wound environment are well known inducers of osteogenic differentiation in MSC cultures (Sidney et al., 2014), and the up-regulation of humoral factors following TBI has been shown to significantly enhance fracture healing and callus formation in patients with long bone defects (Cadosch et al., 2009a). Furthermore, osteogenic differentiation of MSCs exposed to serum extracted from both humans and animals following TBI has established a link between the body's systemic response to injury and HO (Cadosch et al., 2010). Therefore, given the role of inflammatory factors during bone regeneration and the osteoinductive effects of local and systemic factors released in response to trauma on MSCs and skeletal muscle cell differentiation, it seems reasonable to propose that these factors may also play a significant role in the aberrant processes underlying HO.

## Muscle repair and ossification

**Figure 2:**
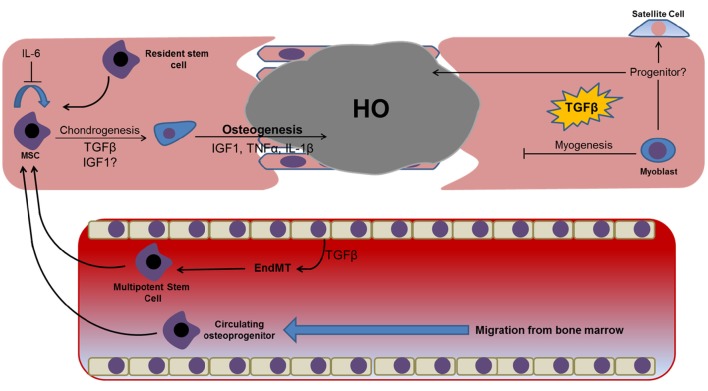
**Prominent factors identified in proteomic and genomic profiling studies of post-trauma serum/exudate and their potential osteochondrogenic effects on cells identified at the wound site**. Dysregulation or prolonged exposure to these factors may prevent efficient wound healing and instead facilitate tissue fibrosis and the osteochondrogenic differentiation of resident and/or migratory cell types. Dysregulation of these factors following traumatic injuries has also been shown to promote the transdifferentiation of resident endothelial cells lining the tissues vasculature, with endothelial-mesenchymal transition representing a well-established example of this phenomenon that has been linked with HO. The presence of a hyper-inflammatory environment may also induce myoblast reversion to a less committed satellite or progenitor cell capable of osteogenic differentiation.

Skeletal muscle trauma and inflammation is accompanied by the activation and migration of resident satellite cells, as well as the breakdown and remodeling of soft tissue at the site of injury. This process contributes to regeneration that serves to replace damaged tissue at the wound site. Every cycle of muscle regeneration leads to an increased deposition of extracellular matrix, specifically interstitial collagen, which is hypothesized to adversely affect regeneration and instead lead to the deposition of fibrous and fatty replacement tissue (Uezumi et al., 2014). It is currently unknown whether this fibrous replacement tissue has a direct relationship with the fibroproliferative lesions preceding HO. However, what is known is that fibrosis becomes exaggerated in the presence of chronic inflammation resulting as a consequence of severe trauma, and that this process appears to be correlated with local production of TGF-β (Mendias et al., 2012). In addition to TGF-β, other prominent growth and inflammatory factors at the site of regenerating skeletal muscle include fibroblast growth factor (FGF), insulin-like growth factors (IGFs), hepatocyte growth factor (HGF) and tumor necrosis factor-alpha (TNF-α). These proteins are secreted by endogenous muscle cells as well as infiltrating leukocytes such as macrophages and neutrophils. Currently, details concerning the contribution of the post-trauma inflammatory response to HO are limited. However, significant attention has been paid to understanding the contribution of persistent inflammatory dysregulation to skeletal muscle breakdown, regeneration, and associated negative outcomes such as tissue fibrosis and fat accumulation. This research will provide a sound basis for understanding how inflammatory dysregulation may contribute toward the misdirected differentiation of stem cells, progenitors and other more committed cells located at the wound site in conditions such as HO. We shall now consider the contribution of several prominent cytokines identified following trauma on skeletal muscle regeneration, and how these cytokines may negatively affect the balance between tissue repair and HO (Figure 2).

TGF-β contributes to muscle fibrosis and healing following injury. This cytokine is largely produced by infiltrating leukocytes, such as neutrophils and macrophages, during the regenerative stage of muscle repair (Figure 1). The effects of TGF-β during embryonic myogenesis are complex and thought to coordinate events leading to myoblast fusion, thereby preventing premature myotube formation (Kollias and McDermott, 2008). The addition of exogenous TGF-β isoforms *in vitro* has been shown to reduce the fusion efficiency of C2C12 myoblasts, leading to a reduction in the total number of myotubes formed (Schabort et al., 2011). Data suggests that this cytokine is likely to exert a concentration-dependent effect when added exogenously to tissue engineered skeletal muscle, with studies showing that 2 ng/ml promotes greater functional and structural characteristics when compared with lower concentrations (Weist et al., 2013). However, tissue engineered models generated in the presence of TGF-β1 in concentrations greater than 2 ng/ml fail to form functional myotubes, suggesting an inhibition of differentiation and maturation. These observations are likely attributed to the specific intra-cellular milieu that largely contributes to the outcome of TGF-β1 in skeletal muscle. Together the data suggest that high levels of TGF-β observed following muscle injury may reduce the total number of terminally differentiated cells, instead causing them to undergo an extended proliferative phase, which is likely required to ensure an adequate number of cells is present at the site of tissue damage before any irreversible myogenic commitment is made.

TGFβ also has a role in directing the expression of scleraxis (Scx) in skeletal muscle fibroblasts, promoting proliferation and collagen type-I synthesis important for tissue repair. This transcription factor is expressed in fibroblasts during embryonic development and recent data suggests that the expression of Scx may have an association with ectopic ossification during tendonopathy (Omachi et al., 2015). However, at present the role of Scx^+^ cells during skeletal muscle ossification remains largely unknown. In addition to the role of TGFβ during skeletal muscle regeneration it has been found to promote activation of the osteogenic transcriptional regulator Runx2 in C2C12 myoblast cultures (Lee et al., 2000). Significantly, the addition of TGF-β1 in combination with BMP-2 caused potent HO *in vivo*, highlighting the possible enhancing properties of this growth factor on BMP-induced ossification (Tachi et al., 2011). Furthermore, the addition of TGF-β1 to primary mouse myoblast cultures has been shown to induce the expression of muscle progenitor cell markers, Pax7 and Sca-1 (Mu and Li, 2011). Together, this data suggests that TGF-β1 is able to induce myoblast reversion to a progenitor-like cell capable of osteogenic differentiation in the presence of osteoinductive factors such as BMP-2. However, we would like to point out that the indirect osteoinductive effects of TGF-β1 are not solely confined to skeletal muscle cells. This cytokine has also been shown to promote endothelial cell trans-differentiation; a process implicated in ectopic bone formation resulting from conditions such as HO and arteriosclerosis (Medici et al., 2010). Together these data highlight TGFβs capacity to contribute to HO either by facilitating osteogenic differentiation in the presence of osteoinductive factors such as BMP-2, or by promoting the transdifferentiation of committed cells to an osteogenic phenotype. Therapies controlling TGFβ levels at the wound site may have considerable value as HO prophylactics, and this may in part explain the efficacy of current NSAID administration.

IGF-1 is released by M2 macrophages that contribute to muscle fiber reconstruction during the anti-inflammatory phase of tissue regeneration (Philippou et al., 2007). Therapeutic application of IGF-1 in preclinical trials has shown increased muscle mass, reduced degeneration and increased satellite cell proliferation (Song et al., 2013). The endogenous production of skeletal muscle IGF-1 is also well documented and plays a role at autocrine and paracrine levels (Adams, 2002). Furthermore, the level of mRNA transcripts specific to IGF-1, are dependent on the extent of original injury intensity (Matheny et al., 2017). IGF-1 signaling has been implicated in many different aspects of muscle regeneration, contributing to protein synthesis and hypertrophy (PI3K/AKT/mTOR pathway) and cell proliferation and mitogenesis (RAS/Raf/MEK pathway) (Song et al., 2013). The production of this anabolic hormone has also been shown to promote the proliferation and migration of satellite cells and inflammatory cells to sites of muscle damage, and blocking either IGF-1 or the IGF-1 receptor *in vivo* leads to grossly underdeveloped skeletal muscle, although the IGF-1 receptor has been shown to be dispensable for post-natal skeletal muscle hypertrophy (Spangenburg et al., 2008). The action of IGF-1 in skeletal muscle is further complicated by a number of IGF-1 binding proteins (IGFBPs) found within the extracellular fluid and function as a depot for the controlled release of IGF-1 (Song et al., 2013); as well as alternative splice variants (Phillippou and Barton, 2014), whereby distinct E-peptides are produced following exercise or injury (Tonkin et al., 2015). Specifically in muscle, three C-terminus peptides are produced from alternative splicing; Ea, Eb, and Ec (also known as MGF in humans; Velloso and Harridge, 2010). These splice-variants contribute to divergent responses, whereby the translated IGF-1ea pro-peptide contributes to differentiation and MGF stimulates proliferation and inhibits differentiation (Ates et al., 2007).

The action of this hormone is not solely myogenic, and there is evidence to suggest that persistently elevated levels of IGF-1 following muscle damage may also be associated with osteochondrogenic differentiation, potentially from the paracrine action of skeletal muscle-derived IGF-1. Indeed, the contribution of IGF-1 to skeletogenesis is well known, with IGF-1 signaling exerting a direct effect on osteoblasts and chondrocytes via an association with growth hormone during embryogenesis (Ahmed and Farquharson, 2010). In addition, IGF-1 also modulates the anabolic effects of parathyroid hormone (PTH), a hormone with important anabolic and catabolic effects during bone remodeling (Lombardi et al., 2011). IGF-1 knockouts were shown to lack the anabolic effects associated with intermittent PTH administration, such as increased alkaline phosphatase activity and greater bone mineral density (Bikle et al., 2002). These effects have, at least in part, implicate IGF-1 signaling in PTH stimulation of RANKL (Wang et al., 2007a) and ephrin B2/EphB4 stimulation of osteoclastogenesis, osteoblast proliferation and differentiation (Matsuo and Otaki, 2012). IGF-1 is particularly important for longitudinal bone growth and defects attributed to IGF-1 gene deletion include extreme short stature in humans (Batey et al., 2014). This hormone also coordinates chondrocyte hypertrophy through Wnt/β-catenin signaling, which represents a critical stage during endochondral ossification; subsequently leading to angiogenesis and remodeling of the cartilaginous matrix to form bone (Wang et al., 2010). A previous study has implicated IGF-1 in the early ossification of bone marrow explants, which is significant given that MSCs may be recruited from the bone marrow to sites of skeletal muscle damage following trauma (Gurkan et al., 2010). Together, these studies highlight the important reparative effects of IGF-1 following skeletal muscle trauma, but also the differential effects that this hormone is likely exert on cells at different stages of lineage commitment. Based on this evidence we suggest that the persistence of IGF-1 in a chronic wound environment could act in conjunction with osteochondrogenic factors to facilitate the formation ectopic bone.

An elevated level of pro-inflammatory cytokines TNF-α and IL-1β is consistent with previous trauma studies (Pasinetti et al., 1993; Evans et al., 2012). These cytokines are primarily associated with the presence of early invading T helper cells and monocytes recruited to the tissue during the initial stages of muscle repair (Rigamonti et al., 2014). Production of these cytokines has also been identified in degenerating and regenerating muscle cells, NK cells, and neutrophils, as well as in endomysial and perimysial connective tissue (Loell and Lundberg, 2010). TNFα persists during the initial reparative stages following muscle injury, facilitating proteolysis and catabolism, and the effects of elevated levels of pro-inflammatory cytokines such as IL1 and TNFα on skeletal muscle wasting have been well documented in a number of inflammatory conditions (Popa et al., 2007). Nevertheless, it appears that endogenous TNFα plays a significant role in skeletal muscle regeneration in a physiological capacity (Chen et al., 2007), while the deleterious effects of this cytokine may be as a consequence of higher concentrations from a paracrine source. Current reports concerning the osteoinductive capacity of these cytokines are mixed. A number of *in vitro* studies have documented osteogenic differentiation in the presence of TNF-α via activation of the NF-KappaB pathway (Hess et al., 2009); while some *in vivo* studies state that this cytokine acts to suppress the osteogenic differentiation of MSCs (Yang et al., 2013). Therefore, it appears likely that the effects of TNF-α on the osteogenic differentiation of stem cells is likely to be dependent on its concentration and association with other inflammatory agents present in complex biological systems. We would suggest that any contribution of TNF-α to HO is perhaps most likely to occur during the chondrogenic phase of differentiation by exerting an osteoinductive effect on multipotent cells at the wound site. This prediction is in line with previous studies showing that the combined presence of pro-inflammatory cytokines TNF-α and IL-1 can promote endochondral ossification in chondrocytes through expression of the pro-osteogenic factor BMP-2, as well as by up-regulating tissue non-specific alkaline phosphatase activity (Lencel et al., 2001; Fukui et al., 2003). Additionally, the presence of these cytokines has been linked with the onset of vascular calcification and the formation of syndesmophytes (ectopic ossifications where ligaments, tendons and capsules attach to bone), thereby proving further links between these factors and ectopic bone formation. Together these reports suggest that the osteochondrogenic effects of TNF-α and IL-1 are highly dependent on the particular stage of ectopic ossification, highlighting that efficient resolution of the pro-inflammatory stage is likely to be important for preventing the effects of osteoinductive factors in the presence of local and migratory progenitor cells.

In addition to IL-1, a number of other interleukins are up-regulated in wound exudate following physical trauma. Of these interleukins, IL-3, -4, -6, and -10 are perhaps the most interesting when one considers muscle regeneration and the pathology of HO. IL-3 is produced by activated T-lymphocytes and contributes to the differentiation of haematopoietic stem cells toward a myeloid lineage. This is significant given that myeloid hyperplasia is a phenomenon associated with HO but paradoxical given that IL-3 is a potent inhibitor of osteoblastic differentiation (Ehrlich et al., 2005). IL-4 is produced by T helper 2 (Th2) cells during tissue regeneration, and associated with an increase in the presence of M2 macrophages, which consequently leads to an increased synthesis of TGF-β and an indirect association with tissue fibrosis (Tatano et al., 2014). Together with other T cell cytokines (IL-1 and TNFα) IL-4 is involved in positively regulating osteoclast maturation through the NF-κB pathway (Hu et al., 2014). The association between IL-4 and osteoclast-mediated bone resorption means that this cytokine is unlikely to contribute toward HO but may instead have a preventative role. IL-6 is released from skeletal muscle following contraction (e.g., exercise), and its autocrine effects on skeletal muscle tissue are equivocal, having been shown to be important for satellite cell mediated muscle growth (Serrano et al., 2008) and implicated in muscle atrophy (Haddad et al., 2005). However, IL-6 is further released by a number of different inflammatory cells following muscle injury, and appears to be important for the chemotactic signaling of macrophages, thereby facilitating muscle regeneration via immune modulation (Zhang et al., 2013). In addition to its roles during muscle regeneration, Il-6 has a complex role in bone homeostasis, and exerts different effects on osteoblasts and osteoclasts depending on their stage of differentiation (Lieder and Sigurjonsson, 2014). Hence this cytokine has been documented as having both resorptive and osteoinductive effects depending on the metabolic state of the cells involved. Notably, IL-6 induces osteogenic differentiation in bone marrow stem cell cultures by down-regulating the expression of Sox2, a transcription factor involved in maintaining stem cell multipotency (Yoon et al., 2014). Lastly, IL-6 can stimulate transcription of the anti-inflammatory cytokine IL-10, which has a central role in regulating macrophage phenotype, promoting a switch to M2 macrophages that are required for normal muscle growth and regeneration (Deng et al., 2012). In the context of bone development, the presence of IL-10 has been shown to be important for endochondral ossification via the stimulation of the BMP signaling pathway (Jung et al., 2013). Importantly, dysregulation in the BMP signaling pathway is the primary cause of FOP, a rare genetic form of HO (Kaplan et al., 2004). Furthermore, there is also substantial evidence to implicate BMP signaling in the development of acquired HO. As such, factors that influence this signaling pathway may represent potential targets for the future therapies.

A number of other proteins with defined roles in muscle regeneration have been shown to contribute to osteogenic differentiation. Amphiregulin, a growth factor secreted by T regulatory (Treg) cells that have been shown to rapidly accumulate within areas of skeletal muscle injury in mice has been shown to contribute to muscle repair *in vivo* (Burzyn et al., 2013). Muscle repair also features two heparin-binding growth factors, HGF and FGF, which are sequestered and presented at the ECM to facilitate regeneration. HGF has recently been shown to be derived from paracrine sources such as activated M2 macrophages but can also be secreted by proliferating satellite cells (Sakaguchi et al., 2014). HGF plays a fundamental role during the early stages of muscle regeneration by stimulating the migration and activation of satellite cells. It has been shown to be released when these cells are mechanically stretched *in vitro* (Tatsumi et al., 2001) and can be observed by *in vivo* time course experiments at 4–6 days following crush injury (Do et al., 2012). Up-regulation of HGF following mechanical stretch or injury may be related to recent discovery that implicates this growth factor in inflammatory resolution, which appears to be linked with inhibition of the inflammatory master regulator, NF-κB/p65 (Proto et al., 2015). In addition to its anabolic role during myogenesis, it has also been demonstrated that binding of HGF to the c-met receptor can act to inhibit the expression of myogenic transcription factors MyoD and myogenin (Anderson, 2016). Recent work has suggested a novel role for HGF in muscle regeneration, whereby selective silencing of muscle-specific HGF contributes to degeneration of the diaphragm in the mdx;p65^±^ model of improved dystrophic phenotype (Proto et al., 2015). Such a response may be as a consequence of the anti-inflammatory effect of HGF. However further work is required to confirm this hypothesis.

Previous studies have also shown that HGF has a concentration-dependent effect on the osteogenic differentiation of MSCs (Aenlle et al., 2014). Consequently, there is a need to understand the potential osteoinductive effects of HGF on resident stem/progenitor cells as well as migrating stem cells from the bone marrow. In addition to HGF, several members of the FGF family of growth factors are released from the cytosol during muscle breakdown and repair. Of this family of proteins, FGF-2 has been shown to have a role in the osteogenic differentiation of MSCs (Miraoui et al., 2009). The action of FGF-2 during muscle repair is not well documented, but appears to promote satellite cell proliferation while repressing myogenic lineage commitment. Therefore, much like physiologically elevated concentrations of TGF-β, FGF-2 may facilitate the production of a pool of resident progenitors that can be osteogenically induced in a post-trauma environment (Jump et al., 2008). Most significantly, it has been proposed that the mechanically induced release of FGF-2 following muscle contraction and plasma membrane disruption represents a potential mechanisms linking the coupled physiological processes of muscle regeneration and bone formation (Hamrick, 2012). Interferon gamma (IFNγ) is another early cytokine frequently identified within the wound effluents of trauma patients. This factor is released by myoblasts and immunological cell types such as macrophages, T cells and natural killer cells in response to tissue damage. IFNγ mediates the hypercatabolic state of multiple cell types following trauma (Madihally et al., 2002), and is one of the principal initiators of M1 macrophage polarization. Furthermore, this cytokine has been identified as an inhibitor of ossification and blocking its expression has been shown to alter myoblast proliferation and fusion (Cheng et al., 2008). The antifibrotic effect of this cytokine is already being exploited to limit the formation of scar tissue following traumas. However, at present the potential prophylactic effects of IFNγ and other early inflammatory cytokines have yet to be examined for patients at risk of developing HO.

In addition to ECM synthesis, efficient muscle repair also requires coordinated tissue breakdown and remodeling. This is primarily the role of a family of calcium-dependent endopeptidases called the matrix metalloproteinases (MMPs) and their tissue inhibitors (TIMPs), which act to breakdown collagen and non-collagenous substrates, allowing the recruitment of myogenic, inflammatory, vascular and fibroblastic cells to the wound bed. The process of tissue breakdown is important as it provides a pathway for myoblast and satellite cell migration and differentiation during muscle repair. The process of is also important for increasing the bioavailability of growth factors sequestered within the ECM. MMPs localized to skeletal muscle during tissue regeneration include MMP-1, 2, 9, and 13, with each likely to have a distinct temporal role during myoblast differentiation (Zimowska et al., 2008). Indeed, distinct roles for MMP-2 and MMP-9 in the migration and differentiation of human skeletal muscle-derived cells have previously been demonstrated by a member of our group (Lewis et al., 2000). Importantly, MMPs degrade fibronectin present in the extracellular matrix to promote myoblast fusion and muscle repair (Chen and Li, 2009). In addition to their roles during muscle repair, MMPs are also highly important for tissue remodeling during endochondral ossification, with the expression of these endopeptidases linked with osteogenic transcription factors. For instance MMP-13 transcription is controlled by Runx2 expression during skeletal embryogenesis and fracture repair (Jimenez et al., 1999). MMP-1 and -2 also have important roles during endochondral bone formation, with allelic mutations in these proteins leading to osteolysis and arthritis-like symptoms (Temtamy et al., 2012). However, with the exception of MMP-9, the roles of these proteins during HO remain largely unknown. MMP-9 is co-localized within polymorphonuclear inflammatory cells such as leucocytes and macrophages that migrate to the site of injury during the inflammatory response. Interestingly, this protein is associated with osteoinductive factors involved in HO, with a study demonstrating MMP-9 co-localization at nerves and vessels as early as 48 h after BMP-treatment. As such it has been hypothesized that this matrixin may represent a diagnostic marker valuable for predicting the onset of HO (Rodenberg et al., 2011).

## Adipogenesis

**Figure 3:**
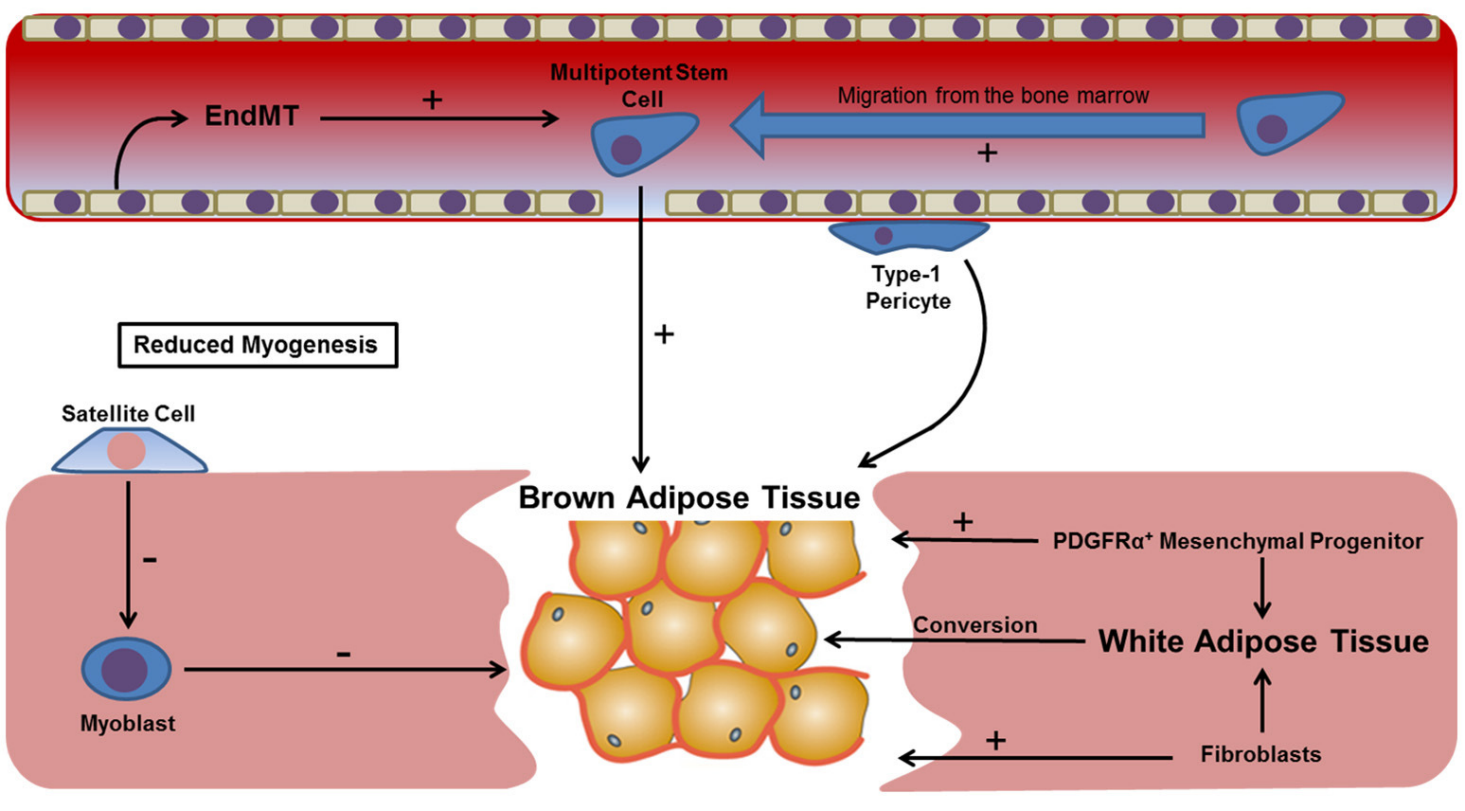
**Potential mechanisms leading to brown adipose tissue formation within regenerating skeletal muscle**. The presence of brown fat following muscle injury establishes a hypoxic gradient that contributes toward a number of processes known to precede HO, including neovascularization and chondrogenesis. However, the origin of this tissue remains unknown, possibly being derived from a number of local progenitors (e.g., satellite cells and type-1 pericytes), migratory cell types (bone marrow MSCs), resident fibroblasts or committed cells shown to undergo transdifferentiation in response to cytokines such as TGF-β. Skeletal muscle satellite cells and myoblasts are not thought to have a significant contribution to the formation of brown fat, with a greater accumulation demonstrated when resident satellite cells are reduced.

Adipocytes are not common within skeletal muscle, and are confined to the connective tissue septa of healthy individuals. The accumulation of fat at sites of muscle fibrosis (fatty degeneration) increases subsequent tissue dysfunction and is linked with failed muscle regeneration. The formation of fat following tissue regeneration also leads to a subsequent decrease in local oxygen tension, which facilitates chondrogenesis and possibly leads to the formation of endochondral bone (Olmsted-Davis et al., 2007). Originally it was proposed that trauma led to dysregulation of the fate switch governing satellite cell differentiation, causing these cells to differentiate to an adipogenic lineage (Shefer et al., 2004). However, subsequent studies revealed that the formation of ectopic fat was actually most significant when muscle regeneration was impaired, such as when the satellite cell population is reduced (Sambasivan et al., 2011). More recent evidence points to the fact that the adipogenic population is likely to be of fibroblast rather than myogenic origin, with the intramuscular injection BMP-2 expressing fibroblasts causing an accumulation of brown adipocytes after just 24 h (Olmsted-Davis et al., 2007). However, in addition to fibroblasts, numerous other endogenous and migratory cell types have shown a capacity for adipogenesis (Davies et al., 2015) (Figure 3). For instance MSCs, hypothesized to migrate from the bone marrow following trauma (Pignolo and Shore, 2013), have a well-defined adipogenic capacity. This is also true of multipotent progenitors localized within skeletal muscle (Lau et al., 2015). Significantly, a population of Sca-1^+^/CD34^+^ fibro/adipogenic progenitors (FAP) have been identified in murine skeletal muscle. These FAP cells are non-myogenic, but proliferate in response to tissue damage, enhancing the rate of proliferation of other myogenic progenitors and facilitating muscle repair. Subsequently, human counterparts have been identified that can be identified by their CD15^+^/PDGFRα^+^/CD56^−^ phenotype (Arrighi et al., 2015). However, since these cells give rise to white adipose tissue rather than brown it is unlikely that they represent the primary contributors to HO. Furthermore, questions regarding the exact relationship between these cells and skeletal muscle fibroblasts remain unanswered owing restrictions in immunophenotyping. However, it should be noted that in the absence of brown fat, white adipocytes have been shown to convert to fat-oxidizing cells, thereby providing a compensatory mechanism for reducing local oxygen tension (Olmsted-Davis et al., 2007). Therefore, the contribution of FAP cells to HO cannot altogether be ruled out.

Skeletal muscle adipogenesis may not be solely confined to cells of mesenchymal origin. Cells of the vasculature may also influence the formation of ectopic adipose tissue at sites of trauma. For instance multipotent cells residing in the vasculature called pericytes have been shown contribute to skeletal muscle adipogenesis. Pericytes can be split into two subpopulations, each with a distinct contribution to skeletal muscle regeneration. Transplant studies have shown that type-2 pericytes have a positive contribution to muscle regeneration, while type-1 pericytes are associated with fatty degeneration (Birbrair et al., 2013). However, the vascular contribution to skeletal muscle adipogenesis may not solely be restricted to multipotent pericytes. Pioneering new research has shown that endothelial cells in adipose tissue capillaries can give rise to both white and brown fat (Tran et al., 2012). This is significant given that a number of studies have identified endothelial-mesenchymal transition (EndMT) as a possible mechanism, at least in part, for the formation of HO (Medici and Olsen, 2012). Based on the wealth of evidence linking aberrant muscle regeneration, fatty degeneration and HO, we propose that blocking adipogenesis may represent an effective way of preventing HO, and it has been highlighted that the use of BMP antagonists may be one way of accomplishing this goal—since BMPs (e.g., BMP-4) have a central role in regulating adipogenic precursor cell commitment and differentiation (Gustafson et al., 2015). The use of BMP antagonists would also likely be useful in limiting the osteochondrogenic differentiation of cells contributing to HO, and we shall explore this in more detail later in the manuscript. Lastly, we would like to end this section by commenting on the seemingly complex relationship between skeletal muscle adipogenesis and the hypoxic tissue environment that is thought to precede HO. Previous studies have shown that hyperglycaemic oxidative stress is able to induce muscle stem cell transdifferentiation, and as such the hypoxic environment following blast trauma is likely to, at least in part, result as a consequence of fatty degeneration during skeletal muscle regeneration (Natalicchio et al., 2011). We shall now explore the association between tissue hypoxia and HO in more detail.

## Tissue hypoxia

**Figure 4:**
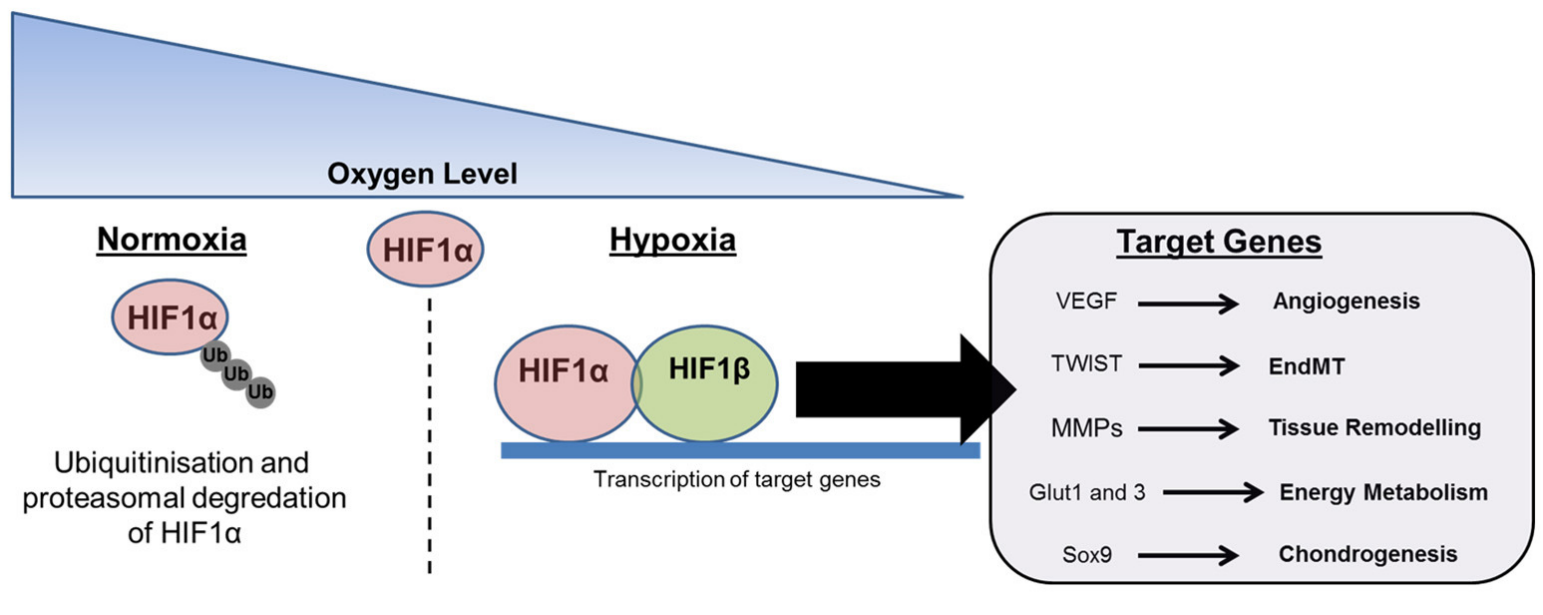
**The effects of oxygen tension on tissue repair and HO**. The presence of tissue hypoxia is frequently described at sites of trauma and has been linked with HO. Under normoxic conditions HIF1α is polyubiquitinated and proteasomally degraded. Under hypoxic conditions HIF1α localizes with HIF1β to form a transcription complex that promotes the synthesis of target genes related to angiogenesis, endothelial-mesenchymal transition (EndMT), tissue breakdown and remodeling, energy metabolism, and chondrogenesis.

Oxygen tension has an important role during wound healing, with either hyperbaric oxygen therapy or low-pressure oxygen therapy routinely used to promote tissue regeneration (Gottrup, 2004). Conversely, low oxygen tension is found to negatively affect tissue regeneration, and the link between local tissue hypoxia and the development of HO is relatively well established (Kan and Kessler, 2011). Low oxygen tension leads to the activation of hypoxia-inducible factor (HIF), a heterodimeric transcription complex that helps coordinate angiogenesis through the induction of vascular endothelial growth factor (VEGF) (Zimmermann et al., 2013) (Figure 4). This process is critical during endochondral bone formation, and has also been associated with cancer development and progression (Cao et al., 2009). Additionally, the presence of HIF1 in arteries has been linked with the development of conditions leading to vascular calcification, such as atherosclerosis (Hao et al., 2014). HIF1 is also important in directing EndMT via regulation of TWIST, a transcription factor implicated in cell determination and differentiation, which is considered an important factor underlying cancer metastasis (Yang and Wu, 2008). This is significant given that EndMT has been proposed as a possible mechanism contributing to HO. Incidentally, given that HO can be defined as pathological ad uncontrolled bone formation at atypical sites, drawing parallels between ectopic bone formation and tumor growth may not be wholly unjustified. HIF1 has also been shown to enhance the expression of MMPs, which breakdown the surrounding matrix, potentially allowing the migration and invasion of a number of inflammatory, vascular and myogenic cell types during muscle repair (Gilkes and Semenza, 2013). Within skeletal muscle HIF contributes to myoblast proliferation and the growth of regenerating myofibres (Scheerer et al., 2013). In this tissue anaerobic ATP and lactate production are increased through glycolysis, and pyruvate dehydrogenase kinase enzymes are upregulated limiting pyruvate decarboxylation and maintaining cellular energy homeostasis at low oxygen concentrations. Energy metabolism has also been shown to be altered through the effects of HIF1α on glucose transport via Glut1 and Glut3 (Mobasheri et al., 2005). Significantly, delivery of echinomycin, a HIF1α inhibitor, in a mouse model was able to significantly reduce the volume of ectopic bone formed following Achilles tenotomy (Zimmermann et al., 2013). Moreover, the HIF1α pathway is important for coupling angiogenesis with osteogenesis during skeletal development (Wang et al., 2007b). An important study that relates HIF to the development of HO has identified that this factor acts as a regulator of BMP2 induced endochondral ossification in fetal limb cultures (Zhou et al., 2015). Additionally, HIF-transduced bone marrow stem cells have also been shown to undergo osteogenic differentiation (Zou et al., 2011). Based on these findings it is likely that reduced oxygen tension at the site of trauma and the accompanying transcription of HIF have a direct contribution to the development of HO. However, blocking or down-regulating HIF is likely to have a negative effect on neo-angiogenesis and tissue regeneration. Since this process represents an essential stage in the regeneration of injured tissues the dose and timing of such a therapy is likely to be critical when treating victims of severe trauma.

## Angiogenesis

The formation of new blood vessels is essential for the regeneration of damaged muscle. However, angiogenesis is also tightly linked with endochondral ossification, and represents an important stage during both embryonic skeletogenesis and fracture callus formation. In a post-trauma environment, neo-angiogenesis in the presence of osteogenic progenitors is likely to facilitate the delivery of cells and soluble factors that contribute to HO. The re-vascularization of damaged tissue is a complex process that relies on the presence of a myriad of different angiogenic factors that are secreted by endogenous myoblasts and muscle progenitors, as well as endothelial cells, pericytes, leukocytes and possibly multipotent cells derived from the circulation. Furthermore, angiogenesis is tightly coupled with myogenesis and has been hypothesized to result from a crosstalk between endothelial cells and resident satellite cells that are located within close proximity to capillaries. Crosstalk between these cells is thought to involve the secretion of soluble factors such as IGF-1, FGF, HGF, and VEGF (Christov et al., 2007). Of these factors, the role of VEGF in tissue revascularization is perhaps best understood. VEGF is expressed by hypertrophic chondrocytes before cartilage becomes vascularized and remodeled to form bone, and also by brown adipocytes found within damaged tissues that go on to develop HO (Ueno et al., 2004). Any link between VEGF and the progression of HO further implicates local or migratory endothelial cells in the process of osteochondrogenic differentiation, which is consistent with histological examination of ectopic bone nodules formed in the soft tissues of patients with ossification (Lounev et al., 2009). These histological examinations noted increased vascularity in ectopic bone, which may be a result of an increased concentration of local VEGF, together with an increase in the proportion of perivascular lymphocytes (Gannon et al., 1997). Increased vascularization during HO development has also been highlighted by Peterson et al. (2014), where it was shown that mice with burn injuries displayed enhanced vascularization within implanted BMP2 transfected ADSC/collagen ossicles (Peterson et al., 2014). Considerable neo-vascularization within ectopic bone suggests the involvement of endothelial cells or migratory mesenchymal stem-like cells with the capacity to form vascular networks. Furthermore, these lymphocytic infiltrates have been shown to overexpress members of the BMP family that are found in high concentration within FOP lesions, and have been shown to induce ectopic bone formation when injected intramuscularly (Leblanc et al., 2011; Grenier et al., 2013).

In addition to the contribution of VEGF during tissue vascularization, platelets that aggregate at the site of local blood vessels also have an important role in directing neo-angiogenesis. The aggregation of platelets at the site of tissue damage initially acts to limit blood loss. However, platelets also have an important role in driving tissue vascularization, with activated platelets releasing PDGF, TGF-β1, VEGF, and bFGF upon degranulation (Torreggiani et al., 2014). The roles of TGF-β1, VEGF, and bFGF have been previously described and we shall not comment on these further. Instead we shall focus our attention in the role of PDGF, which can exist in any of three isoforms: PDGF-AA, -AB, and -BB. Of these three isoforms, only PDGF-BB has been shown to have a significant role during muscle regeneration. As stated, PDGF-BB is initially released by activated platelets during the body's initial response to tissue damage. It is subsequently released by macrophages that enter the tissue via the circulation and promote the migration of muscle precursor cells (Li et al., 2010). Although the exact contribution of PDGF during HO is unknown, recent studies are beginning to outline a clear relationship between PDGF and extra-skeletal ossification. For instance, the addition of platelet-derived exosomes to MSC cultures has been shown to trigger osteogenic differentiation *in vitro* (Torreggiani et al., 2014). Furthermore, a valuable study looking at therapeutic mechanisms for the prevention of HO has identified that the administration of imatinib, a PDGF antagonist, significantly reduced the volume of ectopic bone formed in an Achilles tenotomy model (Werner et al., 2013). As a whole these data suggest that exerting control over the angiogenic response following trauma may represent an effective intervention for reducing HO after trauma, and that PDGF and VEGF represent potential therapeutic targets.

## Endochondral ossification

Ectopic bone formed as a result of HO is frequently described as endochondral (Kan and Kessler, 2011). There is currently a wealth of evidence implicating BMP signaling as a primary mechanism governing the formation of acquired HO. However, the effects of these proteins are not solely confined to osteogenic differentiation, but have also been linked to the physiological regulation of muscle mass (Winbanks et al., 2013). In fact this family of multifunctional growth factors is crucial during embryonic skeletal development, and genetic mutations resulting in alterations in BMP expression can have serious consequences. One such example being FOP, the rare genetic form of HO resulting from an activating missense heterozygous mutation in the ACVR1 gene that encodes the BMP type-I receptor ACVR1/ALK2 (Kaplan et al., 2010). At present our understanding of the mechanisms governing acquired HO are less advanced than for FOP. However, a number of studies have identified that, although these two conditions are pathologically distinct, BMPs play an important role in both forms of the condition.

Of the 20 BMPs currently characterized, previous studies have shown that BMP-2, -6, -7 and -9 can promote osteogenic differentiation in skeletal muscle myoblasts both *in vitro* and *in vivo*, when transplanted into the quadriceps of nude mice (Liu et al., 2009). Furthermore, BMP-4 expressing muscle-derived stem cells undergo osteogenic differentiation *in vivo* (Wright et al., 2002). Administration of commercially available BMP-2 and BMP-7 has been shown to lead to HO at the site of joints and may provide some rationale for the formation of ectopic bone following surgeries such as hip arthroplasty (Axelrad et al., 2008; Chen et al., 2010). In fact, studies examining the effects of BMP-2 on myoblasts and Tie2^+^ PDGFRα^+^ Sca-1^+^ muscle-derived progenitor cells have shown that the presence of BMPs at the site of muscle damage is likely to inhibit myogenesis and promote osteogenic differentiation (Katagiri et al., 1994; Wosczyna et al., 2012). Together these studies lead us to conclude that ectopic bone formed following trauma may result, in part, from the action of BMPs on endogenous muscle progenitors as well as more committed skeletal muscle cells. The role of BMP-2 was perhaps most convincingly demonstrated in a blocking study that utilized recombinant human Gremlin, a BMP-2 antagonist, to reduce calcific tendonopathy, a type of ectopic bone formation affecting one in five people over the age of fifty (Yeung et al., 2014).

We would also like to draw attention to the potential contribution of BMP-2 during neurological HO. This type of HO is frequently associated with TBI or SCI, which can similarly lead to ectopic ossification in tissues such as skeletal muscle (Cipriano et al., 2009). BMP-2 has been linked to the process of sensory nerve induced inflammation by coordinating the release of neuro-inflammatory factors substance P and calcitonin gene-related peptide (CGRP) (Bucelli et al., 2008). The release of neuro-inflammatory factors initiates the recruitment of immunological cells, such as platelets, mast cells and neutrophils to the site of injury and also increases mast cell degranulation and the release of stem cells with an osteochondrogenic potential (Salisbury et al., 2011). Together, this combination of factors is likely to contribute to the misdirected differentiation of multipotent cells toward and osteochondrogenic phenotype and we conclude that there is strong evidence to suggest that BMPs (particularly BMP-2) contribute to HO both directly and indirectly via the peripheral nervous system.

Hormones have a critical role during embryonic skeletogenesis and are involved in the conversion of cartilage to bone during endochondral ossification. Growth hormone (GH) is known to be an important regulator of endochondral bone development by promoting cartilage proliferation, with elevated levels leading to gigantism (Nilsson et al., 2005). This peptide hormone also stimulates the production of IGF-1 and synergizes with BMP-9 to promote chondrocyte and osteoblast activity through the JAK-STAT pathway (Huang et al., 2012). The presence of the hormone-like lipid compound prostaglandin E2 (PGE2) in the urine may be used as in the early diagnosis of HO (Ahrengart, 1991). In fact it is thought that a number of PGs may act as predictors of HO, with these hormones inducing a cAMP cascade hypothesized to represent one possible mechanism governing ectopic bone formation (Bartlett et al., 2006). The relationship between PGE2 and HO formation has even led researchers to examine the efficacy of PGE2-blocking agents such as indomethacin for preventing ectopic bone formation (Schurch et al., 1997). However, further research into this mechanism is required before any firm conclusions can be formulated. Thyroid hormone is another example of a hormone that may have a role in HO development. This hormone acts on chondrocytes to promote hypertrophy and alkaline phosphatase activity, which is critical for cartilage remodeling and ossification during HO (Ballock and Reddi, 1994). Of particular note is the fact that pseudohypoparathyroidism, a condition causing resistance to parathyroid hormone, has been associated with the formation of ectopic bone (Adachi et al., 2009). Interestingly, this condition is caused by a maternally inherited mutation in the GNAS gene. This is of relevance due to the fact that a mutation in GNAS has been shown to activate hedgehog signaling, which has been shown to cause cutaneous ossification in conditions such as progressive osseous heteroplasia (POH), osteoma cutis Albright hereditary osteodystrophy (AHO) (Adegbite et al., 2008; Elli et al., 2013; Regard et al., 2013). As such it is important that the relationship between this hormone and acquired HO is further examined.

## Summary

Acquired HO most frequently occurs as a result of a traumatic insult, such as a blast injury or burns. The progression of this pathology is preceded by inflammatory dysregulation, which in addition to the primary mechanical stimulus, is considered to represent one of the primary factors preceding HO. However, at present there is little experimental data detailing just how the post-trauma inflammatory response can alter the balance between effective muscle repair and adverse pathological outcomes such as HO. Current evidence suggests that there are multiple predisposing factors that include local tissue hypoxia, the bacterial colonization of wounds and the action of pro-angiogenic and osteoinductive factors such as VEGF and BMPs, respectively. All of these stages represent opportunities for clinical intervention. However, we propose that earlier interventions will be most effective, reducing the likelihood of recurrence. We identify that strategies to better understand the contribution of chronic inflammation, and particularly the role of macrophage plasticity in chronic wounds, may be of considerable benefit for the prevention of ectopic bone formation. Currently, NSAIDs are the most commonly prescribed HO prophylactics. However, the efficacy of these anti-inflammatory drugs is limited, suggesting that the timing of delivery is a significant factor governing a satisfactory outcome. This provides further evidence to suggest that the delicate balance between immunosuppressive down-regulation of the inflammatory response and its effects on muscle regeneration and HO need to be further evaluated.

## Author contributions

OD: Concept, design, and drafting of manuscript. LG, ML and YL: Concept and editing. DP and NM: Editing.

## Funding

The work was directly funded by Defense science and technology laboratory (Dstl) and also kindly supported by the EPSRC (Engineering and Physical Sciences Research Council, UK) Centre for Innovative Manufacturing in Regenerative Medicine, The National Centre for Sport and Exercise Medicine (NCSEM) England and FP7-PEOPLE-2012- 0049RSES (SkelGen). OD was supported by a personal EPSRC E-TERM Landscape Fellowship.

### Conflict of interest statement

The authors declare that the research was conducted in the absence of any commercial or financial relationships that could be construed as a potential conflict of interest.
